# Participation in Community Gathering Places and Subsequent Health and Well-Being: An Outcome-Wide Analysis

**DOI:** 10.1093/geroni/igad084

**Published:** 2023-08-11

**Authors:** Kazushige Ide, Atsushi Nakagomi, Taishi Tsuji, Takafumi Yamamoto, Ryota Watanabe, Meiko Yokoyama, Kokoro Shirai, Katsunori Kondo, Koichiro Shiba

**Affiliations:** Department of Social Preventive Medical Sciences, Center for Preventive Medical Sciences, Chiba University, Chiba, Japan; Department of Community General Support, Hasegawa Hospital, Chiba, Japan; Department of Social Preventive Medical Sciences, Center for Preventive Medical Sciences, Chiba University, Chiba, Japan; Department of Cardiology, Chiba University Hospital, Chiba, Japan; Department of Social Preventive Medical Sciences, Center for Preventive Medical Sciences, Chiba University, Chiba, Japan; Institute of Health and Sport Sciences, University of Tsukuba, Tokyo, Japan; Department of Health Promotion, National Institute of Public Health, Saitama, Japan; Department of Social Preventive Medical Sciences, Center for Preventive Medical Sciences, Chiba University, Chiba, Japan; Center for Well-Being Society, Nihon Fukushi University, Aichi, Japan; Department of Social Preventive Medical Sciences, Center for Preventive Medical Sciences, Chiba University, Chiba, Japan; Public Health, Department of Social Medicine, Graduate School of Medicine, Osaka University, Osaka, Japan; Department of Social Preventive Medical Sciences, Center for Preventive Medical Sciences, Chiba University, Chiba, Japan; Department of Gerontological Evaluation, Center for Gerontology and Social Science, Research Institute, National Center for Geriatrics and Gerontology, Aichi, Japan; Department of Epidemiology, Boston University School of Public Health, Boston, Massachusetts, USA

**Keywords:** Cognitive health, Healthy aging, Health behavior, Mental health, Physical health

## Abstract

**Background and Objectives:**

Evidence remains inadequate regarding the benefits of participation in community gathering places, which is Japan’s primary strategy for preventing functional disability in older adults, in other domains of health and well-being. This longitudinal study examined the associations of participation in community gathering places with an array of subsequent health and well-being outcomes among older adults.

**Research Design and Methods:**

We used 3-wave data (2013, 2016, and 2019) from Japan Gerontological Evaluation Study (*n* = 5 879 or 4 232 depending on the outcome). Our exposure was participation in community gathering places in 2016. We assessed 34 health/well-being outcomes in 2019 across 6 domains. We adjusted for pre-baseline covariates including prior outcome values in 2013.

**Results:**

Compared with nonparticipation, participation in community gathering places was associated with some outcomes in the following 3 domains: physical/cognitive health (better higher-level functional capacity), social well-being (more frequent participation in hobby groups, senior citizens clubs, learning or cultural groups, and seeing more friends within a month), and prosocial/altruistic behaviors (more frequent participation in volunteering; after Bonferroni correction as *p* < .0015, .05/34).

**Discussion and Implications:**

Evidence was mixed and more modest for the outcomes in three other domains, mental health, psychological well-being, and health behaviors. Promoting participation in community gathering places may not only fulfill its original goal (ie, preventing functional disability) but also enhance other domains of human well-being, potentially by increasing social interactions.

**Translational Significance:** Promoting participation in community gathering places is the primary strategy for the prevention of functional disability in Japan. Roles of community gathering places in promoting multidimensional health and well-being are understudied. This study demonstrates that participation in community gathering places was associated with some outcomes in the following 3 domains: physical/cognitive health, social well-being, and prosocial/altruistic behaviors. A community intervention with community gathering places may contribute not only to the prevention of functional disability but also to the promotion of health and well-being in some other domains.

Aging populations are ongoing worldwide, and increasingly more countries must tackle the resulting major challenges to ensure that their health and social systems are well prepared for this demographic shift (1). Japan has one of the most aged populations globally (28.8% of the population was aged ≥65 years as of 2020) (2). Hence, Japan’s strategy for handling its aging population can inform future policies in other countries that are expected to face similar problems associated with population aging in the near future (3). Since 2015, Japan has focused on a population-based approach as its primary strategy for the prevention of functional disability (4). Specifically, the Japanese government has promoted community gathering places called “Kayoi-no-ba,” an initiative by local governments to promote social activities and build social capital for the prevention of functional disability (4–7). There are 3 theoretical reasons why building community gathering places might promote healthy aging. First, community gathering places serve as a mutual focal point where locally living older adults can work on health-promoting activities. These activities, which involve physical activities and cognitive exercises, include arts, crafts, music, health education seminar, and physical and brain exercises (4,8). Second, community gathering places may also foster social interaction and build stronger social ties among participants, which can contribute to their health through the exchange of emotional, instrumental, and informational social support, and cultivating community social capital (9,10). Lastly, social engagement through community gathering places may give participants a sense of purpose in life, potentially improving health by buffering psychological distress and promoting health behaviors (11–13). In these community gathering places, local governments, together with citizen volunteers, create social gatherings for older adults, and these gatherings are held in common spaces, such as community centers, neighborhood association halls, and parks that are easily accessible to community members and have a low participation fee (4,7,14). Community gathering places have been widely introduced across 95.9% (1 670/1 741) of Japanese municipalities in 2019 and participated by 6.7% (2 374 726/35 486 813) of the entire older adult population (15).

Participation in community gathering places is associated with various health-related outcomes, including physical function (16), physical activity (17), social participation (18), self-rated health (19), instrumental activities of daily living (ADL) (18), intellectual activities (18), frailty (20,21), functional disability (22–24), dementia (25), and medical costs (17). However, these previous studies have faced several challenges. First, most previous studies only examined a single (19–25) or a few outcomes (16–18) at a time. Health is not simply the absence of disease, but a multidimensional construct defined as “a state of complete physical, mental, and social well-being.” (26) However, some important domains of such multidimensional well-being (eg, purpose in life) are understudied. Promoting social capital via community gathering places might backfire—for example, it may increase smoking, alcohol consumption, and excessive stress due to peer pressure (27); hence, examining wide-ranging outcomes simultaneously will help us evaluate the comprehensive and holistic effects of community gathering places on multiple domains of health and well-being. Second, the study design of the prior works could have been improved from causal inference perspectives. For example, most of the prior studies relied on repeated cross-sectional design (20) or longitudinal design with only 2 survey waves (17,18,23) or design using data from only the group participating in the community gathering place (16,21). In a cross-sectional study, causality cannot be inferred because of reverse causation (ie, prebaseline health affects participation in community gathering places). Adjusting for outcome values before exposure assessment (ie, participation in community gathering places) can be effective in reducing reverse causality and bias due to some types of unmeasured confounders (28). However, such rigorous adjustment has rarely been done because it requires data from at least 3 waves (1 for preexposure covariates, 1 for exposure, and 1 for outcome assessment).

Therefore, the present study examined the longitudinal associations between participation in a community gathering place and wide-ranging subsequent health and well-being outcomes among older adults in Japan. We leveraged 3-wave data from a nationwide cohort study of Japanese older adults and adjusted for preexposure covariates, including preexposure outcome values, to reduce bias due to confounding and reverse causation. We adopted an outcome-wide approach (29) and holistically estimated the effects of participation in community gathering places on wide-ranging outcomes from the following domains: physical/cognitive health, mental health, subjective well-being, social well-being, pro-social/altruistic behaviors, and health behaviors.

## Method

### Study Participants

**Figure 1.**
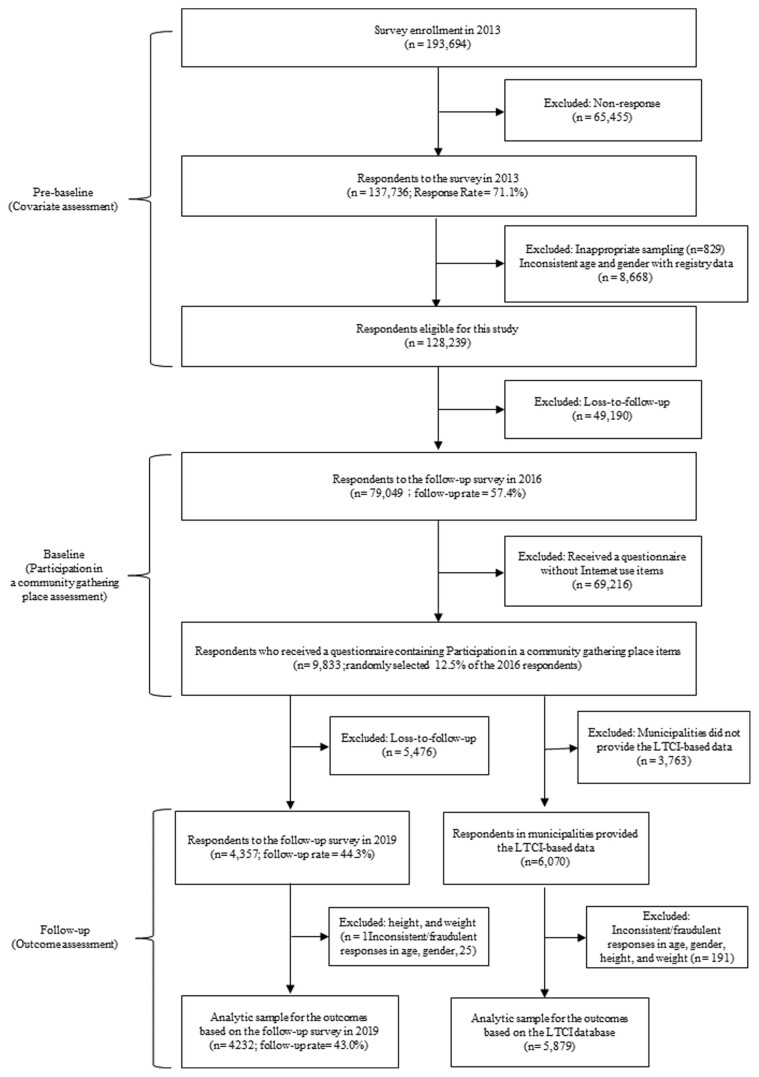
Participant flow for analytic sample. LTCI = Long-term care insurance.

We used 3-wave data from the 2013, 2016, and 2019 surveys of the Japan Gerontological Evaluation Study (JAGES) (30,31), a nationwide survey of older adults without national long-term care insurance (LTCI) service in Japan. Figure 1 shows a detailed flowchart of participant selection, and Supplementary Figure 1 illustrates the temporal order of data linkage and the variables included in the analysis. In 2013, JAGES mailed self-administered questionnaires to physically and cognitively independent older adults aged 65 years and over (*n* = 137 736; response rate: 71.1%). In 2016, JAGES conducted a follow-up survey (*n* = 79 049, follow-up: 57.4%). JAGES randomly selected 12.5% (*n* = 9 833) of the respondents and distributed a questionnaire containing items regarding participation in community gathering places. These 9 833 individuals were categorized into 2 samples by linking them to 1) the 2019 follow-up survey containing information on self-reported outcomes (*n* = 4 357; follow-up rate: 44.3%) and 2) the national LTCI database containing information on the onset of all-cause mortality, dementia, and functional disability between 2016 and 2019 (*n* = 6 070). We excluded respondents with inconsistently reported age or gender between survey waves and improbable height (<100 cm or >200 cm) or weight values (<30 kg or >100 kg). Ultimately, we included 4 232 individuals for the 2019 survey-based outcomes and 5 879 individuals for the LTCI-based outcomes.

This study was performed in line with the principles of the Declaration of Helsinki. Ethical approval for this study was obtained from the Ethics Committee at Chiba University (Approval number: 2493) and the National Center for Geriatrics and Gerontology (Approval number: 992). JAGES respondents were explained that participation in the study was voluntary and that completing and returning the self-administered questionnaire meant agreeing to participate in the study.

### Measures

#### Exposure variable

Our exposure variable was participation in a community gathering place, taken from the 2016 survey. This variable was measured using the following question: *“How long have you been participating in salon activities at local governments and social welfare councils?”* Participants were given the following answer choices: *“never participated,” “used to participate but stopped,” “participated less than 1 year,” “1 year to less than 2 years,” “2 years to less than 3 years,” “3 years to less than 4 years,” “more than 4 years,”* and *“participating but for an unknown period.”* We categorized the responses “*never participated*” and “*used to participate but stopped*” as not participating and the other responses as participating in community gathering places.

#### Outcome variables

Building on theorizing around multidimensional well-being and following a prior outcome-wide study in Japan, we examined 34 outcomes across 6 domains of health and well-being in 2019 (3 years after the exposure assessment) (32–34). These domains included the physical/cognitive health, mental health, subjective well-being, social well-being, pro-social/altruistic behaviors, and health behaviors. In particular, physical/cognitive health domain consisted of death, cognitive disability (dementia), functional (physical or cognitive) disability, natural remaining teeth, self-rated health, body mass index (BMI), higher-level functional capacity (35,36), and self-reported current treatment for chronic diseases (hypertension, diabetes, dyslipidemia, heart disease, stroke, and respiratory disease). The mental health domain included depressive symptoms and a sense of hopelessness. The subjective well-being domain included happiness and life satisfaction. Social well-being domain included frequency of participation in sports group/hobby group/senior citizens club/learning or cultural groups, frequency of meeting friends, number of friends, frequency of going out, emotional social support, and instrumental social support. Prosocial/altruistic behaviors domain included frequency of volunteering and sharing of skills and experiences. Finally, health behaviors domain consisted of current smoking, meat and fish intake, vegetable and fruit intake, walking time, and participation in health screening.

Data on death, cognitive disability (dementia), and functional (physical or cognitive) disability during the 3-year follow-up were obtained by linking the participants to registries maintained by local municipal governments recorded under the Japanese LTCI system (37,38). Cognitive and functional disability levels were identified by eligibility assessment for LTCI benefits; a trained investigator evaluated applicants requesting for long-term care in terms of ADL and instrumental ADL (IADL), cognitive function, and mental and behavioral disorders following a standardized protocol and assessed whether they were eligible to receive benefits (see Supplemental Text, Supplementary Table 1 and Supplementary Material for more details). Except for the outcomes from the LTCI database (ie, death, dementia, and functional disability), data on all other outcomes were obtained from the 2019 survey. Supplementary Table 2 shows further details about each outcome measurement.

### Covariates

We obtained all covariates from the 2013 survey—3 years prior to the 2016 survey in which the exposure was assessed. We included the following preexposure characteristics: age, gender, years of education (≤9, 10–12, or ≥13 years), household equivalized income (continuous value), employment (never, retired, or current), marital status (married, single, or others), living alone, ADL (dependent or independent), and population density (continuous value). In identifying the population density of habitable land, we divided the population by the area of habitable land at the municipality level from national statistical data (39,40). These characteristics were potential confounding factors that could correlate with participation in community gathering places and the various subsequent health and well-being outcomes. More specifically, we expect that community gathering places participants will be female and healthier compared to non-participants based on the findings of previous studies (22,25).

To reduce the possibility of reverse causation (ie, preexposure health and well-being affect participation in community gathering places), we also adjusted for prior values of the outcomes, except for death, dementia, and functional disability as the participants did not have these conditions in the preexposure wave by design.

### Statistical Analysis

All continuous values are expressed as mean (standard deviation (*SD*)), and categorical variables are reported as numbers (percentages). We did not statistically compare covariates. Statistical tests are not appropriate to assess the presence of confounding (41). We showed the prebaseline characteristics and prior outcome values taken from the 2013 study sample linked to the 2019 survey (*n* = 4 232, Table 1) and linked to national LTCI records (*n* = 5 879; Supplementary Table 3)

**Table 1.** Prebaseline Characteristics and Prior Outcome Values Taken From 2013 Stratified By Participation in a Community Gathering Place in 2016 of the Study Sample Linked to the 2019 survey (*n* = 4 232)^a^

Prebaseline Characteristics	Participation in a Community Gathering Place			
	Nonparticipation *n* = 3 263		Participation *n* = 658	
	mean (SD)	n (%)		
Sociodemographic factors				
Age (years)	71.8 (5.0)		72.3 (4.9)	
Gender (female)		1 593 (48.8)		439 (66.7)
Education				
≤9 years		1 040 (31.9)		175 (26.6)
10–12 years		1 344 (41.2)		315 (47.9)
≥13 years		833 (25.5)		165 (25.1)
Household income (10 thousand yen)	257.1 (162.3)		243.4 (132.3)	
Employment				
Never		305 (9.4)		60 (9.1)
Retired		1,865 (57.2)		456 (69.3)
Current		901 (27.1)		106 (16.1)
Marital status				
Married		2,553 (78.2)		492 (74.8)
Single/others		678 (20.8)		159 (24.2)
Living alone		366 (11.2)		89 (13.5)
Activities of daily living (independent)		3163 (96.9)		638 (89.0)
Population density (per square km)	4,394.4 (3,612.8)		4,043.7 (3,520.8)	
Prior physical/cognitive health				
No natural teeth remaining		210 (6.4)		35 (5.3)
Self-rated health (excellent and good)		2,859 (87.6)		583 (88.6)
Body mass index (kg/m^2^)	22.9 (3.1)		22.9 (3.0)	
Higher-level functional capacity	11.8 (1.6)		12.3 (1.1)	
Self-reported hypertension		1,345 (41.2)		247 (37.5)
Self-reported diabetes		399 (12.2)		72 (10.9)
Self-reported dyslipidemia		469 (14.4)		114 (17.3)
Self-reported heart disease		294 (9.0)		57 (8.6)
Self-reported stroke		73 (2.2)		12 (1.8)
Self-reported respiratory disease		134 (4.1)		25 (3.8)
Prior mental health				
Depressive symptoms	2.7 (3.0)		2.1 (2.5)	
Hopeless		430 (13.2)		64 (9.7)
Prior psychological well-being				
Happiness	7.5 (1.7)		7.6 (1.6)	
Life satisfaction		2,728 (83.6)		580 (88.2)
Prior social well-being				
Participation in sports group	2.0 (1.6)		2.8 (1.8)	
Participation in hobby group	2.1 (1.5)		3.1 (1.5)	
Participation in senior citizens club	1.2 (0.7)		1.7 (1.1)	
Participation in learning or cultural groups	1.3 (0.8)		1.8 (1.2)	
Frequency of meeting friends	3.7 (1.6)		4.3 (1.4)	
Number of friends seen within a month	3.6 (1.3)		4.1 (1.1)	
Frequency of going out	5.7 (0.6)		5.8 (0.5)	
Emotional social support		3,053 (93.6)		631 (95.9)
Instrumental social support		3,085 (94.5)		623 (94.7)
Pro-social/altruistic behaviors				
Volunteering	1.4 (1.0)		2.1 (1.4)	
Sharing skills and experiences	1.3 (0.9)		1.5 (1.0)	
Prior health behaviors				
Current smoking status		334 (10.2)		26 (4.0)
Frequency of meat and fish intake	5.3 (1.1)		5.4 (1.1)	
Frequency of vegetable and fruit intake	6.1 (1.0)		6.4 (0.8)	
Walking	2.4 (1.0)		2.4 (1.0)	
Health screening		2,101 (64.4)		459 (69.8)

*Notes*: SD = standard deviation.

^a^Study sample linked to the 2019 survey (*n* = 4 232).

We adopted an outcome-wide analytic approach (29,32). This approach examines the effects of a single exposure on multiple outcomes, thereby providing holistic evidence on exposure-health associations and reducing the risk of p-hacking and publication bias (29). This approach has been applied in several studies, including 2 studies conducted in Japan in which internet use (33) and having a purpose in life (“Ikigai”) (13) were exposures of interest. We applied separate regression models to examine the associations between participation in community gathering places taken from the 2016 survey and the various outcomes taken from the 2019 survey, adjusting for both preexposure characteristics and prior outcome values taken from the 2013 survey (Supplementary Figure 1). Depending on the nature of the outcome, we used different models: (1) logistic regression for rare binary outcomes with <10% prevalence (death, dementia, functional disability at level 2, no natural teeth remaining, stroke, respiratory disease, and current smoking); (2) Poisson regression for nonrare binary outcomes with a prevalence >10% (functional disability at any levels, self-rated health, hypertension, diabetes, dyslipidemia, heart disease, hopelessness, life satisfaction, emotional social support, instrumental social support, and health screening); and (3) linear regression for continuous outcomes (BMI, higher-level functional capacity, depressive symptoms, happiness, participation in a sports group/hobby group/ senior citizens club/learning or cultural groups, frequency of meeting friends, number of friends seen within a month, volunteering, sharing of skills and experiences, vegetable and fruit intake, and walking). Logistic regression models for rare binary outcomes yield odds ratios approximating risk ratios, whereas modified Poisson regression models for nonrare binary outcomes estimate risk ratios (42). All continuous outcomes were standardized (mean = 0, *SD* = 1) to allow the effect estimates to be interpreted as SD changes in the corresponding outcomes. Standard errors were clustered at the municipality district level to determine the potential correlation of the participants within the same district.

To address potential issues of multiple testing and increased risk for false positives resulting from the simultaneous assessment of the associations between participation in community gathering places and the 34 outcomes (outcome-wide analytic approach), we used Bonferroni correction (29). In this method, we divided the nominal significance level of the test (α = .05) by the number of tests and defined a more conservative *p*-value cutoff for Bonferroni correction as *p* = .0015 (0.05/34).

The self-administered questionnaires contained missing data; hence, using multivariate normal imputation, we created 20 imputed data and combined the estimates across imputations by using Rubin’s rule (43,44).

We also conducted 2 additional analyses. To evaluate the robustness of the estimated associations to unmeasured confounding, we calculated *E*-values for each exposure-outcome association (45). *E*-values quantified the unmeasured confounder’s minimum required strength of association on the risk ratio scale with both the exposure and outcome, above and beyond the adjusted covariates, to explain the observed association. Moreover, we examined the antecedents of participation in community gathering places by conducting a modified Poisson regression analysis with robust standard errors to estimate the risk ratios for the associations between the preexposure characteristics and participation in community gathering places. All statistical analyses used Stata 17/IC (StataCorp, College Station, TX, USA).

## Results

Table 1 shows the prebaseline characteristics and prior outcome values taken from the 2013 study sample linked to the 2019 survey (*n* = 4 232). Community gathering places participants were more likely to be female, have lower household incomes, be unemployed, and be single compared with nonparticipants. Overall, community gathering places participants reported better physical/cognitive health, except for self-rated health, BMI, and dyslipidemia in the prebaseline wave. They also had fewer prebaseline depressive symptoms and sense of hopelessness, higher life satisfaction with subjective well-being, higher social well-being (excluding frequency of going out), more pro-social/altruistic behaviors (ie, volunteering and sharing of skills and experiences), and better health behaviors (except for walking and frequency of meat and vegetable consumption). Similar trends were found for prebaseline characteristics and prior outcome values in the study sample linked to national LTCI records (*n* = 5 879; Supplementary Table 3).

Table 2 shows the estimated standardized beta coefficients (continuous outcomes), risk ratios (nonrare binary outcomes), and odds ratios (rare binary outcomes) for the association of participation in community gathering places with health and well-being in 2019, adjusting for preexposure characteristics and prior outcome values taken from the 2013 survey. Community gathering place participants had better higher-level functional capacity (standardized beta = 0.13; 95% confidence interval [CI]: 0.08, 0.19; *p* < .001), were involved in a hobby group more frequently (standardized beta = 0.25; 95% CI: 0.17, 0.34; *p* < .001), participated in senior citizens club more frequently (standardized beta = 0.30; 95% CI: 0.20, 0.39; *p* < .001), participated in learning or cultural groups more frequently (standardized beta = 0.24; 95% CI: 0.12, 0.37; *p* < .001), had greater number of friends seen within a month (standardized beta = 0.21; 95% CI: 0.12, 0.29; *p* < .001), and participated in volunteering groups more frequently(standardized beta = 0.36; 95% CI: 0.26, 0.46; *p* < .001) in 2019 than nonparticipants. After Bonferroni correction, these associations remained below the *p* = .05 threshold. Compared with nonparticipation, participation in community gathering places was moderately associated with low diabetes incidence, low depression scores, better frequency of participation in a sports group, frequency of going out, frequency of meeting friends, frequency of participation in sharing of skills and experiences, health screening, and frequency of fruit and vegetable consumption. However, these associations were above the threshold of *p* = .05 after Bonferroni correction. There was little evidence of association between participation in community gathering places and other outcomes such as death, dementia, or functional disability.

**Table 2.** Participation in a Community Gathering Place in 2016 and Subsequent Health and Well-being in 2019 Among Older People in Japan

Outcomes in 2019	Participation in a Community Gathering Place					
	Nonparticipation	Participation				
	Reference	RR/OR/β	95% CI		*p* Value	
Physical/cognitive health						
Death	1.00	1.14	0.80	1.61	.465	
Dementia	1.00	1.00	0.50	2.02	.998	
Functional disability (any levels)	1.00	1.22	0.96	1.56	.103	
Functional disability (level 2 or greater)	1.00	1.08	0.73	1.59	.705	
No natural teeth remaining	0.00	0.80	0.48	1.33	.384	
Self-rated health	1.00	1.01	0.98	1.05	.451	
Body mass index	0.00	0.00	−0.05	0.05	.982	
Higher-level functional capacity	0.00	0.13	0.08	0.19	<.001	***
Self-reported hypertension	1.00	1.03	0.94	1.12	.578	
Self-reported diabetes	1.00	0.86	0.74	0.99	.040	*
Self-reported dyslipidemia	1.00	1.08	0.92	1.27	.338	
Self-reported heart disease	1.00	0.90	0.75	1.09	.290	
Self-reported stroke	1.00	1.08	0.61	1.90	.801	
Self-reported respiratory disease	1.00	0.83	0.49	1.39	.473	
Mental health						
Depressive symptoms	0.00	−0.10	−0.18	−0.03	.006	**
Hopelessness	1.00	0.82	0.66	1.01	.061	
Psychological well-being						
Happiness	0.00	0.06	−0.02	0.14	.130	
Life satisfaction	1.00	1.03	1.00	1.06	.054	
Social well-being						
Participation in sports group	0.00	0.12	0.03	0.21	.008	**
Participation in hobby group	0.00	0.25	0.17	0.34	<.001	***
Participation in senior citizens club	0.00	0.30	0.20	0.39	<.001	***
Participation in learning or cultural groups	0.00	0.24	0.12	0.37	<.001	***
Frequency of meeting friends	0.00	0.10	0.03	0.18	.005	**
Number of friends seen within a month	0.00	0.21	0.12	0.29	<.001	***
Frequency of going out	0.00	0.08	0.02	0.15	.011	*
Emotional social support	1.00	1.01	1.00	1.03	.110	
Instrumental social support	1.00	1.00	0.98	1.02	.832	
Pro-social/altruistic behaviors						
Volunteering	0.00	0.36	0.26	0.46	<.001	***
Sharing skills and experiences	0.00	0.13	0.03	0.23	.009	**
Health behaviors						
Current smoking status	1.00	0.96	0.53	1.76	.905	
Frequency of meat and fish intake	0.00	0.04	−0.03	0.12	.238	
Frequency of vegetables and fruits intake	0.00	0.10	0.04	0.16	.002	**
Walking	0.00	0.04	−0.03	0.11	.245	
Health screening	1.00	1.07	1.00	1.14	.035	*

*Notes*: CI = confidence interval; OR = odds ratio; RR = risk ratio.

^a^All continuous outcomes (body mass index, higher-level functional capacity, depressive symptoms, happiness, participation in sports group, participation in hobby group, participation in senior citizens club, participation in learning or cultural groups, frequency of meeting friends, number of friends seen within a month, volunteering, sharing skills and experiences, eating meat and fish, eating vegetables and fruits, and walking) were standardized (mean = 0, standard deviation = 1), and β was the standardized effect size.

The estimates for the rare binary outcomes (no natural teeth remaining, stroke, respiratory disease, and current smoking) were odds ratios estimated by logistic regression. The estimates for other dichotomized outcomes (self-rated health, hypertension, diabetes, dyslipidemia, heart disease, hopelessness, life satisfaction, emotional social support, instrumental social support, and health screening) were risk ratios estimated by modified Poisson regression.

^b^ All models were controlled for sociodemographic factors (age, gender, education, household income, employment, marital status, living alone, and population density), baseline activities of daily living and prior outcome values except for death, dementia, and functional disabilities.

^c^ Regression was performed using the study sample linking the 2013 and 2016 surveys to the national long-term care insurance data (*n* = 5 879) for the outcomes of death, dementia, and functional disabilities and using the study sample linking the 2013 and 2016 surveys to the 2019 survey (*n* = 4 232) for all other outcomes.

* *p* < .05 before Bonferroni correction; ** *p* < .01 before Bonferroni correction; *** *p* < .05 after Bonferroni correction (the *p*-value cutoff for Bonferroni correction is *p* = .05/34 outcomes = *p* < .0015).

Table 3 shows the calculated *E*-values, which indicated that the observed associations between participation in community gathering places and some outcomes were robust to an unmeasured confounder. For example, for the association between participation in community gathering places and hobby group participation, an unmeasured confounder would need to be associated with both the exposure and outcome by a risk ratio of 1.83-fold each (conditional on the measured covariates) to fully explain the observed association and by that of 1.62-fold to shift the CI to include the null value.

**Table 3.** Robustness to Unmeasured Confounding (*E*-values) of Associations Between Participation in a Community Gathering Place and Subsequent Health and Well-Being in 2019 in Japan.

Outcomes in 2019	*E*-values	
	Point Estimate	CI Limit
Physical/cognitive health		
Death	1.54	1.00
Dementia	1.03	1.00
Functional disability (any levels)	1.75	1.00
Functional disability (level 2 or greater)	1.37	1.00
No natural teeth remaining	3.56	1.00
Self-rated health	1.13	1.00
Body mass index	1.02	1.00
Higher-level functional capacity	1.50	1.35
Self-reported hypertension	1.19	1.00
Self-reported diabetes	1.61	1.09
Self-reported dyslipidemia	1.38	1.00
Self-reported heart disease	1.45	1.00
Self-reported stroke	1.36	1.00
Self-reported respiratory disease	1.72	1.00
Mental health		
Depressive symptoms	1.43	1.20
Hopelessness	1.75	1.00
Psychological well-being		
Happiness	1.30	1.00
Life satisfaction	1.19	1.00
Social well-being		
Participation to sports group	1.47	1.21
Participation to hobby group	1.83	1.62
Participation to senior citizens club	1.95	1.70
Participation to learning or cultural groups	1.80	1.48
Frequency of meeting friends	1.43	1.20
Number of friends seen within a month	1.71	1.49
Frequency of going out	1.37	1.16
Emotional social support	1.12	1.00
Instrumental social support	1.05	1.00
Prosocial/altruistic behaviors		
Volunteering	2.12	1.85
Sharing skills and experiences	1.50	1.21
Health behaviors		
Current smoking status	1.23	1.00
Frequency of meat and fish intake	1.24	1.00
Frequency of vegetables and fruits intake	1.42	1.23
Walking	1.24	1.00
Health screening	1.35	1.07

*Note:* CI = confidence interval.

Table 4 shows the results of the antecedent analysis. We found that older age, female, better higher-level functional capacity, higher social well-being (frequency of participation in a hobby group/senior citizens club/ learning or cultural groups), and more prosocial and altruistic behaviors (frequency of volunteering) in the preexposure wave predicted subsequent participation in community gathering places. The highest risk ratio was observed for gender (1.49), which was smaller than the *E*-value for hobby group participation (1.62).

**Table 4.** Antecedents of Participation in a Community Gathering Place in 2016.

Prebaseline Characteristics	Community Gathering Places Participation in 2016				
	RR	95%CI		*p* Value	
Sociodemographic factors					
Age	1.02	1.01	1.03	.001	***
Female (vs. male)	1.49	1.25	1.79	<.001	***
Education (vs. ≤9 years)					
10–12 years	1.16	1.04	1.29	.007	**
≥13 years	1.07	0.94	1.22	.297	
Household income (yen)	1.00	1.00	1.00	.076	
Employment (vs. current)					
Retired	1.32	1.08	1.61	.006	**
Never	1.02	0.79	1.31	.886	
Married (vs. single/others)	1.01	0.83	1.22	.950	
Living alone (vs. living not alone)	1.04	0.85	1.28	.674	
Activities of daily living independent (vs. not independent)	1.05	0.79	1.40	.718	
Population density (per 100 square km)	0.99997	0.99995	0.99999	.003	**
Physical/cognitive health					
No natural teeth remaining	0.80	0.62	1.03	.081	
Self-rated health	0.82	0.69	0.98	.030	**
BMI	1.02	0.96	1.07	.539	
Higher-level functional capacity	1.16	1.06	1.26	.001	***
Self-reported hypertension	0.91	0.82	1.02	.098	
Self-reported diabetes	1.03	0.86	1.23	.742	
Self-reported dyslipidemia	0.95	0.81	1.12	.558	
Self-reported heart disease	0.99	0.82	1.19	.882	
Self-reported stroke	0.88	0.56	1.40	.595	
Self-reported respiratory disease	1.02	0.82	1.28	.839	
Mental health/psychological distress					
Depressive symptoms	0.99	0.91	1.08	.885	
Hopelessness	1.07	0.89	1.29	.473	
Psychological well-being					
Happiness	1.00	0.93	1.07	.959	
Life satisfaction	0.93	0.77	1.13	.473	
Social well-being					
Participation to sports group	1.07	1.01	1.14	.028	*
Participation to hobby group	1.12	1.05	1.19	.001	***
Participation to senior citizens club	1.18	1.13	1.23	<.001	***
Participation to learning or cultural groups	1.10	1.06	1.15	<.001	***
Frequency of meeting friends	1.02	0.95	1.09	.587	
Number of friends seen within a month	1.12	1.04	1.21	.005	**
Frequency of going out	0.98	0.93	1.04	.554	
Emotional social support	0.86	0.66	1.12	.270	
Instrumental social support	0.81	0.60	1.09	.168	
Character and virtue					
Volunteering	1.20	1.15	1.26	<.001	***
Sharing skills and experiences	0.97	0.92	1.01	.128	
Health behaviors					
Current smoking status	0.82	0.62	1.09	.181	
Frequency of meat and fish intake	0.91	0.86	0.97	.002	**
Frequency of vegetables and fruits intake	1.15	1.05	1.25	.002	**
Walking	0.04	-0.03	0.11	.245	
Health screening	0.95	0.90	1.01	.131	

*Notes*: CI = confidence interval; RR = risk ratio.

^a^ We used a modified Poisson regression with robust standard errors to estimate prevalence ratios for the association between each of the predictors in 2013 and participation in Community gathering places in 2016, controlling for all other prebaseline covariates.

* *p* < .05 before Bonferroni correction; ** *p* < .01 before Bonferroni correction; *** *p* < .05 after Bonferroni correction (the *p*-value cutoff for Bonferroni correction is *p* = .05/34 outcomes = *p* < .0015).

## Discussion

This longitudinal study with a 3-year follow-up period examined the relationship between participation in community gathering places, which is the primary strategy for the prevention of functional disability in Japan, and subsequent health and well-being. There are 5 main findings. First, participation in community gathering places was associated with several social well-being outcomes, such as more frequent participation in a hobby group/senior citizens club/learning or cultural groups and a greater number of friends seen within a month. Second, participation in community gathering places was associated with more frequent volunteering. Third, in terms of physical/cognitive health, community gathering place participants had better higher-level functional capacity than nonparticipants. Fourth, there was modest evidence that participation in a community gathering place was associated with lower depressive symptoms scores and a higher frequency of fruit and vegetable consumption. Fifth, there was no strong evidence that participation in community gathering places was associated with other measures of subsequent health and well-being.

The observed associations between participation in community gathering places and outcomes in social well-being domain are consistent with a previous study (18) wherein a year after the opening of a community gathering place, participants had more opportunities to get involved in sports group than nonparticipants. With a 3-year follow-up period, the present study extended the prior evidence and demonstrated that community gathering place participants (vs. nonparticipants) were involved more frequently in more diverse types of social activities, including hobby group, senior citizens club, and study or cultural groups. A possible explanation for this finding is that community gathering place participants may, through interactions with other participants at the gathering, get invited to or receive information on other activities that take place outside of the gathering. This finding is in line with the government’s aim to promote social interaction and build social capital among older community-dwelling adults through a community gathering place (4,5,7).

Similarly, volunteering, which was included in the pro-social and altruistic behavior domain, might be promoted by participation in community gathering places. Volunteers perceive that participation offers opportunities close at hand (46). In a survey conducted by the National Council of Social Welfare, 20.2% of the respondents answered that they participated in volunteer activities because they were “invited by a friend or acquaintances” (47).

In terms of the outcomes in the physical/cognitive health domain, community gathering place participants had better higher-level functional capacity than nonparticipants, and this result is consistent with the original aim of the community-based interventions (ie, prevention of functional disability) and the findings of a prior prospective cohort study (18). In our study, the indicators of higher-level functional capacity included instrumental self-maintenance (eg, “Can you go out alone by train or bus?”), intellectual activities (eg, “Are you interested in health-related articles or TV programs?”), and social roles (eg, “Do you give advice to family and friends?”) (35), which can be facilitated by participation in community gathering places. Observed associations, if causal, suggest that participating in a community gathering place may facilitate older adults to use a train or bus or to go out with people they meet at the gathering more often. Similarly, conversations at community gathering places may increase interest in health-related information and provide more opportunities to visit friends’ homes or offer advice to friends.

We did not find evidence of an association between participation in community gathering places and mortality, dementia onset, and functional disability during the 3-year follow-up period even though these outcomes are primary targets of the prevention of functional disability in Japan (4,7). In previous studies (22,25), the establishment of social interaction and the maintenance of physical and cognitive functions through participation in community gathering places inhibited functional disability and dementia onset. The inconsistency may be due to the more rigorous adjustment of confounding and reverse causation that we performed. It is also possible that our follow-up length was too short for the protective effects of participation in community gathering places on functional disability, dementia, and mortality to manifest. In past studies, the follow-up periods were approximately 4 (23), 5 (22), and 6 (24) years for functional disability and 7 years (25) for dementia. A previous study reported no difference in functional disability between participants and nonparticipants in a 2-year follow-up, but a difference was detected in a 4-year follow-up (23). Several prior studies (48–51) on social participation and mortality also had a minimum follow-up period of 7.4 years (48) and maximum of 20 years (51). Further studies with a longer follow-up period are warranted to examine the role of community gathering places in preventing functional and cognitive disabilities.

Contrary to our findings, a previous study examined a similar study population of older adults in Japan and reported a significant association between participation in community gathering places and improvement in self-rated health by utilizing instrumental variable estimation (19). This inconsistency may be attributable to differences in target populations and a lack of variance. The instrumental variable approach estimates the average compliers effect—a subpopulation of individuals who adheres the treatment status that was indicated by the instrument (52,53). In contrast, our study examined the exposure effects of the entire study sample. If the compliers and the remaining individuals in older population in Japan differ in terms of characteristics that could contribute to effect heterogeneity, the results may not be comparable.

Although the association was above the threshold of *p* = .05 after Bonferroni correction, our study showed the modest association of participation in community gathering places with lower depressive symptoms scores. Considering the robust evidence in the associations between social participation in older people and depression (54), and assuming that the relationship observed in this study is causal, our study suggests that participation in a community gathering places may alleviate depressive symptoms directly and/or indirectly via promoted social interaction and social participation as we found in this study.

Among the health behaviors, the frequency of fruit and vegetable intake tended to be higher among community gathering places participants, although this finding needs to be interpreted with caution because the association was above the threshold of *p* = .05 after Bonferroni correction. A previous study reported that local social networks may promote frequent consumption of fruits and vegetables (55). This mechanism may be explained by social contagion, which refers to the notion that information and behaviors spread through a social network (9). Some older adults may obtain health information and be encouraged to consume a healthy diet (eg, fruits and vegetables) as a result of participating in a community gathering place.

Although participation in community gathering places seems effective, the antecedent analysis revealed some challenges in its future dissemination. Community gathering places participants were mostly females, older, and unemployed. Particularly, female predominance in the participation in community gathering places is consistent with several prior studies in Japan (5,16–19,21–25). In Japan, the proportion of working older adults is particularly high among males (56). In addition, many males do not participate in community organizations because they are too busy (57). Offering attractive programs for retired males may be a key to promoting male participation. The participation rate of older males was reported to be higher in community gathering places with some specific programs (eg, hobbies and exercise programs) (5). Recruiting males as staff to run the community gathering place may be effective because males feel proud to have a role (58). Because popular programs likely differ by gender and other factors, local governments may need to consider what type of programs they offer to attract more diverse participants.

An outcome-wide approach provides a holistic assessment of a single exposure for a wide range of outcomes as discussed earlier (29,32). Recently, several studies (13,33) explored the associations between an exposure of interest and various outcomes by leveraging a 3-wave data set. A study in Japan, for example, used the same data set as this study and explored the multidimensional impacts of internet use on health and well-being for the promotion of internet usage among older adults (33). The outcome-wide approach can be a potential tool to provide evidence for the implementation and promotion of an intervention of interest, which was participation in a community gathering place in this study.

Our study has some limitations. First, we cannot exclude the possibility of unmeasured confounding (eg, household wealth). Nonetheless, we adjusted for a rich set of covariates, including prior levels of outcomes, taking advantage of the 3-wave panel structure. Moreover, our sensitivity analysis using *E*-value suggested that some evidence in this study is robust even considering the unmeasured confounding. For example, the *E*-value for the 95% CI limit of hobby group participation was 1.62. This suggests that bias due to an unmeasured confounder even as strong as gender, which was the strongest predictor of participation in community gathering places conditional on other observed covariates (risk ratio = 1.49), cannot explain the observed association. In addition, we ensured that covariates were observed before the exposure assessment, so the over-adjustment of potential mediators is unlikely. Second, reverse causation cannot be avoided completely. For example, older adults who participated in community gathering places originally had a high level of functionality, resulting in participation in community gathering places. However, for this reverse causality, we took advantage of the 3-wave panel structure, which enables us to include prior outcome levels to reduce. Such control for baseline outcome does not eliminate the possibility of reverse causation but helps to mitigate it (29). Third, selection bias due to selective attrition is possible. The largest sample attrition in this study was the random selection of participants receiving the questionnaire containing the community gathering place item in the 2016 survey (12.5% of the original sample). This attrition was random; hence, the resulting selection bias is likely minimal. However, attrition in other steps of the sample selection (eg, loss-to-follow-up between baseline and the follow-up wave) might have caused selection bias. Our post hoc analysis indicated that the analytic sample linking the 2013 wave, the 2016 wave, and the 2019 wave (*n* = 4 232) tended to be younger, more educated, and married and report higher income as well as better health/well-being in the prebaseline wave, compared with the sample of the data linking the 2013 wave and the 2016 wave (*n* = 7 612) and the analytic sample linking the 2013 wave, the 2016 wave, and the national long-term care insurance record (*n* = 5 879) (Supplementary Table 4). Fourth, the types of activities (eg, hobbies, exercise, or intellectual activities) offered at community gathering places could not be considered because such data was not available in this study. The types of activities most effective in promoting health and well-being need to be explored in the future, including an understanding of which older adults participate in which activities.

In conclusion, this study showed a wide range of evidence that participation in community gathering places promotes the health and well-being of older adults in Japan. Participation in community gathering places was associated with several social well-being outcomes, such as more frequent participation in a hobby group/senior citizens club/learning or cultural groups and a greater number of friends seen within a month. Furthermore, participation in community gathering places was associated with more frequent volunteering. Additionally, in terms of physical/cognitive health, participation in community gathering places had better higher-level functional capacity than nonparticipants. A community intervention with community gathering places, which is the primary strategy for the prevention of functional disability in Japan, may contribute not only to the prevention of functional disability but also to promoting other domains of human well-being by increasing social interactions. With restrictions on face-to-face contact during the coronavirus disease 2019 (COVID-19) pandemic, community gathering places were forced to suspend activities. After COVID-19-related deregulation, community gathering places will play a major role in restoring face-to-face interaction among older adults.

## Supplementary Material

igad084_suppl_Supplementary_Material
