# Supplementary material for: Participation in Community Gathering Places and Subsequent Health and Well-Being: An Outcome-Wide Analysis
Source: Innov Aging. 2023 Aug 11;7(9):igad084. doi: 10.1093/geroni/igad084 (PMC10724175; doi:10.1093/geroni/igad084)

**Online Supplementary Material**

**Supplemental text on assessment of cognitive** **disability levels**

Cognitive disability grade was categorized into eight levels: 0, I, IIa, IIb, IIIa, IIIb, IV, and M where a higher level indicates increasing severity (M: requires specialized medical care) (Supplementary Table 1). This cognitive impairment scale strongly correlated with Mini-Mental State Examination scores (Spearman’s rank correlation r=−0.73, p < 0.01) ^1^, and level I of the scale corresponded with a 0.5 point rating on the Clinical Dementia Rating scale (specificity and sensitivity were 0.88) ^2^. Dementia onset was defined as level II or higher versus level 0 or 1 (binary variable) ^3^.

Functional disability was defined as being dependent in ADL by physical or cognitive difficulty (as described above) identified by certification for any levels (including the “need support” level) or level 2 or greater in LTCI scheme^4^.

1. Shinya K. The relationship between Revised Hasegawa Dementia Scale (HDS‐R), Mini‐Mental State Examination (MMSE) and Bed‐fast Scale, dementia scale (In Japanese). *Jpn J Geriatr Psychiatry* 2009; 20: 883-891.

2. Meguro K, Tanaka N, Kasai M, et al. Prevalence of dementia and dementing diseases in the old-old population in Japan: the Kurihara Project. Implications for Long-Term Care Insurance data. *Psychogeriatrics* 2012; 12: 226-234. 2013/01/03. DOI: 10.1111/j.1479-8301.2012.00406.x.

3. Noda H, Yamagishi K, Ikeda A, et al. Identification of dementia using standard clinical assessments by primary care physicians in Japan. *Geriatr Gerontol Int* 2018; 18: 738-744. 2018/01/18. DOI: 10.1111/ggi.13243.

4. Ministry of Health, Labour and Welfare. Long-term Care Insurance in Japan., <https://www.mhlw.go.jp/english/topics/elderly/care/2.html> (2021, accessed July 28 2021).

Supplementary Table 1 Cognitive disability grade in the long-term care insurance system in Japan

| Rank | | Criteria |
| --- | --- | --- |
| I |  | Having some dementia-related symptoms, but the daily living is almost independent inside and outside the home. |
| II |  | Having some dementia-related symptoms, behavioral disturbance and/or communication difficulties that limit the daily activities but can be independent under someone’s care. |
|  | IIa | The abovementioned conditions in II are observed only outside the home. |
|  | IIb | The abovementioned conditions in II are observed even inside the home. |
| III |  | Occasionally having dementia-related symptoms, behavioral disturbance and/or communication difficulties that limit the daily activities and require care. |
|  | IIIa | The abovementioned conditions in III are observed predominantly during the day. |
|  | IIIb | The abovementioned conditions in III are observed predominantly at night. |
| IV |  | Frequently having dementia-related symptoms, behavioral disturbance and/or communication difficulties that limit the daily activities and constantly require care. |
| M |  | Having significant mental symptoms, problematic behaviors, or severe physical illnesses that require specialized medical care. |

Supplementary Table 2 Definition of outcome variables

| Outcomes by Theme | | Original Variable Name in JAGES | Original Item Question and Response Options | | | Outcome Definition |
| --- | --- | --- | --- | --- | --- | --- |
| 1. Physical/cognitive health | |  |  | | |  |
|  | Death | eve_sibou | Data were from the long-term care insurance database. All-cause deaths through 2019 were recorded. | | | Binary (1 = death during the three-year follow-up period, 0 = alive) |
|  | Dementia | eve_dem | Data were from the long-term care insurance database. The cognitive disability was assessed at participants' homes by trained investigators dispatched from the certification committee in each municipality. All dementia onset through 2019 were obtained. | | | Binary (1 = dementia during the three-year follow-up period, 0 = no dementia) 5 |
|  | Functional disability (Any levels) | eve_rc0 | Data were from the long-term care insurance database. Functional disability was assessed both through computer-based and home-visit interviews with trained health professionals. All functional disabilities onset through 2019 were obtained. | | | Binary (1 = functional disability during the three-year follow-up period, 0 = no functional disability) |
|  | Functional disability (Level 2 or greater) | eve_rc2 |  |  |  |  |
|  | No natural teeth remaining | teeth5_19 | Questions: “How many natural teeth do you presently have?” | | | Binary (1 = zero remaining vs. 0 = at least one remaining) |
|  |  |  | Options: | | |  |
|  |  |  |  | | 1. I have no natural teeth. |  |
|  |  |  |  | | 2. I have 1-9 natural teeth. |  |
|  |  |  |  | | 3. I have 5-9 natural teeth. |  |
|  |  |  |  | | 4. I have 10-19 natural teeth. |  |
|  |  |  |  | | 5. I have 20 or more natural teeth. |  |
|  | Self-rated health | srh_4_19 | Question: “How is your current health status?” | | | Binary (1=excellent and good vs. 0=fair and poor) |
|  |  |  | Options: | | |  |
|  |  |  |  | | 1. Excellent |  |
|  |  |  |  | | 2. Good |  |
|  |  |  |  | | 3. Fair |  |
|  |  |  |  | | 4. poor |  |
|  | Body mass index | wghtz_19, hghtmz_19, hghtcz_19 | Questions: “What is your current height and weight?” | | | Continuous |
|  |  |  | Formula: wghtz_19/(hghtmz_19+hghtcz_19/100)^2^ | | |  |
|  | Higher-level functional capacity | iadl3bt_19 - iadl2ty19_01 | Questions: | | | Continuous (Range: 0-13, 13 indicate the greatest independence) |
|  |  |  |  | | 1. Can you go out alone by train or bus? |  |
|  |  |  |  | | 2. Can you go shopping for daily necessities? | • Sum of the 13 items {Koyano, 1991 #144} |
|  |  |  |  | | 3. Can you cook for yourself? |  |
|  |  |  |  | | 4. Can you pay your bills by yourself? |  |
|  |  |  |  | | 5. Can you deposit or withdraw money from your bank/postal savings account(s) by yourself? |  |
|  |  |  |  | | 6. Can you complete paperwork for your pension or other reasons by yourself? |  |
|  |  |  |  | | 7. Do you read newspapers? |  |
|  |  |  |  | | 8. Do you read books or magazines? |  |
|  |  |  |  | | 9. Are you interested in health-related articles or TV programs? |  |
|  |  |  |  | | 10. Do you visit your friends’ homes? |  |
|  |  |  |  | | 11. Do you give advice to your family members or friends? |  |
|  |  |  |  | | 12. Can you visit people who have fallen ill? |  |
|  |  |  |  | | 13. Do you start conversations with young people? |  |
|  |  |  | Options: | | |  |
|  |  |  |  | | 1. Yes (or Yes but I usually don’t) |  |
|  |  |  |  | | 0. No |  |
|  | Self-reported hypertension | dgns2ht19 | Questions: "“Did you have the following diseases for which you are currently receiving treatment or experiencing after-effects?-Hypertension | | | Binary (1=hypertension vs. 0=not hypertension) |
|  |  |  | Options: | | |  |
|  |  |  |  | | 0. No |  |
|  |  |  |  | | 1. Yes |  |
|  | Self-reported diabetes | dgns2dm19 | Questions: "“Did you have the following diseases for which you are currently receiving treatment or experiencing after-effects?-Diabetes | | | Binary (1=diabetes vs. 0=not diabetes) |
|  |  |  | Options: | | |  |
|  |  |  |  | | 0. No |  |
|  |  |  |  | | 1. Yes |  |
|  | Self-reported dyslipidemia | dgns2hl19 | Questions: "“Did you have the following diseases for which you are currently receiving treatment or experiencing after-effects?-Dyspidemia | | | Binary (1=dyslipidemia vs. 0=not dyslipidemia) |
|  |  |  | Options: | | |  |
|  |  |  |  | | 0. No |  |
|  |  |  |  | | 1. Yes |  |
|  | Self-reported heart disease | dgns2hd19 | Questions: "“Did you have the following diseases for which you are currently receiving treatment or experiencing after-effects?-Heart disease | | | Binary (1=heart disease vs. 0=not heart disease) |
|  |  |  | Options: | | |  |
|  |  |  |  | | 0. No |  |
|  |  |  |  | | 1. Yes |  |
|  | Self-reported stroke | dgns2st19 | Questions: "“Did you have the following diseases for which you are currently receiving treatment or experiencing after-effects?-Stroke | | | Binary (1=stroke vs. 0=not stroke) |
|  |  |  | Options: | | |  |
|  |  |  |  | | 0. No |  |
|  |  |  |  | | 1. Yes |  |
|  | Self-reported respiratory disease | dgns2rd19 | Questions: "“Did you have the following diseases for which you are currently receiving treatment or experiencing after-effects?-Respiratory disease | | | Binary (1=respiratory disease vs. 0=not respiratory disease) |
|  |  |  | Options: | | |  |
|  |  |  |  | | 0. No |  |
|  |  |  |  | | 1. Yes |  |
| 2. Psychological distress | |  |  |  | |  |
|  | Depressive symptoms | gds_2sf19 – gds_2oc19 | The Japanese version of Geriatric Depression Scale (15 items). | | | Continuous (range: 0-15), greater values indicate more depressive symptoms |
|  |  |  |  | |  |  |
|  | Hopelessness | gds_2nh19 | Question: “Do you think there is no hope in your life?” | | | Binary (1=hopeless vs. 0=hope) |
|  |  |  | Options: | | |  |
|  |  |  |  | | 1. Yes |  |
|  |  |  |  | | 0. No |  |
| 3. Subjective well-being | |  |  |  | |  |
|  | Happiness | happy11_19 | Question: “To what degree do you feel you are currently happy?” | | | Continuous (range: 0-10), 10 indicates very happy |
|  |  |  | Option: | | |  |
|  |  |  | 0-10 (0 for very unhappy and 10 for very happy) | | |  |
|  | Life satisfaction | gds_2sf19 | Question: “Are you satisfied with your current life?” | | | Binary (1=satisfied vs. 0=not satisfied) |
|  |  |  | Option: | | |  |
|  |  |  |  | | 1. Yes |  |
|  |  |  |  | | 0. No |  |
| 4. Social well-being | |  |  |  | |  |
|  | Participation in sports group | cmnt6hb19 | Question: “How often do you attend activities for the following groups – Sports groups” | | | Continuous (1-6, 1 indicates no attendance) |
|  |  |  | Options: | | |  |
|  |  |  |  | | 1. 4 or more times a week |  |
|  |  |  |  | | 2. 2 -3 times a week |  |
|  |  |  |  | | 3. Once a week |  |
|  |  |  |  | | 4. 1-3 times a month |  |
|  |  |  |  | | 5. A few times a year |  |
|  |  |  |  | | 6. Never |  |
|  | Participation in hobby group | cmnt6sp19 | Question: “How often do you attend activities for the following groups – Hobby groups or clubs” | | | Continuous (1-6, 1 indicates no attendance) |
|  |  |  | Options: | | |  |
|  |  |  |  | | 1. 4 or more times a week |  |
|  |  |  |  | | 2. 2 -3 times a week |  |
|  |  |  |  | | 3. Once a week |  |
|  |  |  |  | | 4. 1-3 times a month |  |
|  |  |  |  | | 5. A few times a year |  |
|  |  |  |  | | 6. Never |  |
|  | Participation in senior citizens club | cmnt6sg19 | Question: “How often do you attend activities for the following groups – Senior citizens club” | | | Continuous (1-6, 1 indicates no attendance) |
|  |  |  | Options: | | |  |
|  |  |  |  | | 1. 4 or more times a week |  |
|  |  |  |  | | 2. 2 -3 times a week |  |
|  |  |  |  | | 3. Once a week |  |
|  |  |  |  | | 4. 1-3 times a month |  |
|  |  |  |  | | 5. A few times a year |  |
|  |  |  |  | | 6. Never |  |
|  | Participation in learning or cultural groups | cmnt6le19 | Question: “How often do you attend activities for the following groups – Learning or cultural groups” | | | Continuous (1-6, 1 indicates no attendance) |
|  |  |  | Options: | | |  |
|  |  |  |  | | 1. 4 or more times a week |  |
|  |  |  |  | | 2. 2 -3 times a week |  |
|  |  |  |  | | 3. Once a week |  |
|  |  |  |  | | 4. 1-3 times a month |  |
|  |  |  |  | | 5. A few times a year |  |
|  |  |  |  | | 6. Never |  |
|  | Frequency of meeting friends | meet6fr19 | Question: “How often do you see your friends?” | | | Continuous (1-6, 1 indicate hardly/none) |
|  |  |  | Options: | | |  |
|  |  |  |  | | 1. 4 or more times a week |  |
|  |  |  |  | | 2. 2 -3 times a week |  |
|  |  |  |  | | 3. Once a week |  |
|  |  |  |  | | 4. 1-3 times a month |  |
|  |  |  |  | | 5. A few times a year |  |
|  |  |  |  | | 6. Hardly/None |  |
|  | Number of friends seen within a month | num5fr19 | Question: “How many friends/acquaintances have you seen over the past month? Count the same person as one, no matter how many times you have seen him/her.” | | | Continuous (1-5, 1 indicate none) |
|  |  |  | Option: | | |  |
|  |  |  |  | | 1. None |  |
|  |  |  |  | | 2. 1-2 |  |
|  |  |  |  | | 3. 3-5 |  |
|  |  |  |  | | 4. 6-9 |  |
|  |  |  |  | | 5. 10 or more |  |
|  | Frequency of going out | gout6fq19 | Question: "How often do you go out?" | | | Continuous (1-6, 1 indicates rarely) |
|  |  |  |  | | 1. 4 or more a week |  |
|  |  |  |  | | 2. Two or three times a week |  |
|  |  |  |  | | 3. Once a week |  |
|  |  |  |  | | 4. One to three times a month |  |
|  |  |  |  | | 5. Several times a year |  |
|  |  |  |  | | 6. Rarely |  |
|  | Emotional social support | lsnd2no19 | Question: “Do you have someone who listens to your concerns and complaints? – I do not  have such a person” | | | Binary (1=I do have such a person) vs. |
|  |  |  | Option: | | | 0=I do not have such a person) |
|  |  |  |  | | 0. I do not have such a person |  |
|  |  |  |  | | 1. I do have such a person |  |
|  | Instrumental social support | card2no19 | Question: “Do you have someone who looks after you when you are sick and confined to a bed for a few days? – I do not have such a person” | | | Binary (1=I do have such a person) vs. |
|  |  |  | Option: | | | 0=I do not have such a person) |
|  |  |  |  | | 0. I do not have such a person |  |
|  |  |  |  | | 1. I do have such a person |  |
| 5. Pro-social/altruistic behaviors | | |  |  | |  |
|  | Volunteering | cmnt6vl19 | Question: “How often do you attend activities for the following groups – Volunteer group” | | | Continuous (1-6, 1 indicates no attendance) |
|  |  |  | Options: | | |  |
|  |  |  |  | | 1. 4 or more times a week |  |
|  |  |  |  | | 2. 2 -3 times a week |  |
|  |  |  |  | | 3. Once a week |  |
|  |  |  |  | | 4. 1-3 times a month |  |
|  |  |  |  | | 5. A few times a year |  |
|  |  |  |  | | 6. Never |  |
|  | Sharing skills and experiences | cmnt6sk19 | Question: “How often do you attend activities for the following groups – Activities to teach skills or pass on experiences to others” | | | Continuous (1-6, 1 indicates no attendance) |
|  |  |  | Options: | | |  |
|  |  |  |  | | 1. 4 or more times a week |  |
|  |  |  |  | | 2. 2 -3 times a week |  |
|  |  |  |  | | 3. Once a week |  |
|  |  |  |  | | 4. 1-3 times a month |  |
|  |  |  |  | | 5. A few times a year |  |
|  |  |  |  | | 6. Never |  |
| 6. Health behaviors | |  |  |  | |  |
|  | Current smoking status | smok5_19 | Question: “Do you smoke cigarettes?” | | | Binary (1=current smoker vs. 0=non-smoker) |
|  |  |  | Options: | | |  |
|  |  |  |  | | 1. I smoke almost every day. |  |
|  |  |  |  | | 2. I sometiems smoke. |  |
|  |  |  |  | | 3. I quit smoking within 5 years and do not smoke now. |  |
|  |  |  |  | | 4. I quit smoking more than 5 years ago and do not smoke now. |  |
|  |  |  |  | | 5. I never smoke. |  |
|  | Eating meat and fish |  | Question: "How often did you eat meat and fish over the past month?" | | | Continuous (1-7, 1 indicates none) |
|  |  |  |  | | 1. Twice a day or more |  |
|  |  |  |  | | 2. Once a day |  |
|  |  |  |  | | 3. Four to sic times a week |  |
|  |  |  |  | | 4. Two of three times a week |  |
|  |  |  |  | | 5. Once a week |  |
|  |  |  |  | | 6. Less than once a week |  |
|  |  |  |  | | 7. None |  |
|  | Eating vegetables and fruits |  | Question: "How often did you eat fruits and vegetables over the past month?" | | | Continuous (1-7, 1 indicates none) |
|  |  |  |  | | 1. Twice a day or more |  |
|  |  |  |  | | 2. Once a day |  |
|  |  |  |  | | 3. Four to sic times a week |  |
|  |  |  |  | | 4. Two of three times a week |  |
|  |  |  |  | | 5. Once a week |  |
|  |  |  |  | | 6. Less than once a week |  |
|  |  |  |  | | 7. None |  |
|  | Walking | walk4tm19 | Question: "How long do you walk a day on average?" | | | Continuous (1-4, 1 indicates less than 30 minutes) |
|  |  |  |  | | 1. Less than 30 minutes |  |
|  |  |  |  | | 2. 30 to 59 minutes |  |
|  |  |  |  | | 3. 60-89 minutes |  |
|  |  |  |  | | 4. 90 minutes or more |  |
|  | Physical exam screening | exam4_19 | Question: “Have you ever had a check-up at a health center, your workplace, a medical institution, or another place?” | | | Binary (1=within a year vs. 0=not within a year) |
|  |  |  | Option: | | |  |
|  |  |  |  | | 1. I had one within a year. |  |
|  |  |  |  | | 2. I had one sometime between 1 and 4 years ago. |  |
|  |  |  |  | | 3. I had one more than 4 years ago. |  |
|  |  |  |  | | 4. I’ve never had one. |  |

Supplementary Table 3 Pre-baseline characteristics and prior outcome values taken from 2013 stratified by participation in a Community gathering places in 2016 of the study sample linked to the national long-term care insurance record (n = 5,879)^a^

| Pre-baseline characteristics | | Participation in a community gathering place | |
| --- | --- | --- | --- |
|  |  | Nonparticipation | Participation |
|  |  | n=4,443 | n=930 |
| Sociodemographic factors | |  |  |
|  | Age (years), mean (SD) | 72.6 (5.5) | 73.6 (5.3) |
|  | Gender (Female), n (%) | 2,261 (50.9) | 637 (68.4) |
|  | Education, n (%) |  |  |
|  | ≤9 years | 1,711 (38.5) | 320 (34.4) |
|  | 10–12 years | 1,742 (39.2) | 417 (44.8) |
|  | ≥13 years | 913 (20.6) | 180 (19.4) |
|  | Household income (million yen), mean (SD) | 241.5 (158.1) | 243.4 (142.4) |
|  | Employment, n (%) |  |  |
|  | Never | 459 (10.3) | 97 (10.4) |
|  | Retired | 2,570 (57.8) | 630 (67.7) |
|  | Current | 1,100 (24.7) | 137 (14.7) |
|  | Marital status, n (%) |  |  |
|  | Married | 3,374 (75.6) | 654 (70.3) |
|  | Single/others | 1,008 (22.7) | 260 (28.0) |
|  | Living alone, n (%) | 519 (11.7) | 72 (14.2) |
|  | Activities of daily living (independent), n (%) | 4,282 (96.5) | 897 (96.5) |
|  | Population density (per square kilometer), mean (SD) | 3086.7 (3050.7) | 2070.3 (2779.3) |
| Prior physical/cognitive health | |  |  |
|  | No natural teeth remaining, n (%) | 358 (8.1) | 56 (6.0) |
|  | Self-rated health (excellent and good), n (%) | 3,785 (88.6) | 797 (85.7) |
|  | Body mass index (kg/m2), mean (SD) | 23.0 (3.3) | 23.0 (3.2) |
|  | Higher-level functional capacity, mean (SD) | 11.7 (1.7) | 12.2 (1.2) |
|  | Self-reported hypertension, n (%) | 1,903 (42.8) | 382 (41.1) |
|  | Self-reported diabetes, n (%) | 573 (12.9) | 108 (11.6) |
|  | Self-reported dyslipidemia, n (%) | 609 (13.7) | 139 (15.0) |
|  | Self-reported heart disease, n (%) | 429 (9.7) | 88 (9.5) |
|  | Self-reported stroke, n (%) | 124 (2.8) | 20 (2.2) |
|  | Self-reported respiratory disease, n (%) | 200 (4.5) | 45 (4.8) |
| Prior mental health | |  |  |
|  | Depressive symptoms, mean (SD) | 3.0 (3.1) | 2.4 (2.6) |
|  | Hopelessness, n (%) | 678 (15.3) | 109 (11.7) |
| Prior psychological well-being | |  |  |
|  | Happiness, mean (SD) | 7.3 (1.8) | 7.6 (1.7) |
|  | Life satisfaction, n (%) | 3,642 (82.0) | 803 (86.3) |
| Prior social well-being | |  |  |
|  | Participation in sports group, mean (SD) | 1.8 (1.5) | 2.6 (1.8) |
|  | Participation in hobby group, mean (SD) | 2.0 (1.5) | 2.9 (1.6) |
|  | Participation in senior citizens club, mean (SD) | 1.3 (0.8) | 1.8 (1.2) |
|  | Participation in learning or cultural groups, mean (SD) | 1.3 (0.8) | 1.8 (1.2) |
|  | Frequency of meeting friends, mean (SD) | 3.7 (1.6) | 4.3 (1.4) |
|  | Number of friends seen within a month, mean (SD) | 3.5 (1.3) | 4.0 (1.1) |
|  | Frequency of going out, mean (SD) | 5.7 (0.7) | 5.7 (0.6) |
|  | Emotional social support, n (%) | 4,128 (92.9) | 889 (95.6) |
|  | Instrumental social support, n (%) | 4,168 (93.8) | 871 (93.6) |
| Pro-social/altruistic behaviors | |  |  |
|  | Volunteering, mean (SD) | 1.4 (0.9) | 2.0 (1.3) |
|  | Sharing skills and experiences, mean (SD) | 1.2 (0.8) | 1.4 (1.0) |
| Prior health behaviors | |  |  |
|  | Current smoking status, n (%) | 447 (10.7) | 48 (5.2) |
|  | Frequency of meat and fish intake, mean (SD) | 5.3 (1.1) | 5.3 (1.1) |
|  | Frequency of vegetables and fruits intake, mean (SD) | 6.1 (1.0) | 6.4 (0.8) |
|  | Walking, mean (SD) | 2.4 (1.1) | 2.4 (1.0) |
|  | Health screening, n (%) | 2,771 (62.4) | 650 (69.9) |

Abbreviations: SD, standard deviation.

Supplementary Table 5 Comparison of pre-baseline characteristics and prior outcome values among the respondents to the 2013 and 2016 surveys (n = 8,433), the analytic sample linked to the 2019 surveys (n = 4,232), and the analytic sample linked to the national long-term care insurance record (n = 5,879)

| Pre-baseline characteristics and prior outcomes | | Respondents to the 2013 and 2016 survey | Analytic sample | |
| --- | --- | --- | --- | --- |
|  |  |  | Linked to the 2019 surveys | Linked to the national long-term care insurance record |
|  |  | n=8,433 | n=4,232 | n=5,879 |
| Sociodemographic factors | |  |  |  |
|  | Age (years), mean (SD) | 72.9 (5.5) | 72.0 (5.0) | 72.9 (5.5) |
|  | Gender (Female), n (%) | 4,113 (54.0) | 2,202 (52.0) | 3,200 (54.4) |
|  | Education, n (%) |  |  |  |
|  | ≤9 years | 2,789 (36.6) | 1,336 (31.6) | 2,262 (38.5) |
|  | 10–12 years | 2,989 (39.3) | 1,767 (41.8) | 2,321 (39.5) |
|  | ≥13 years | 1,692 (22.2) | 1,069 (25.3) | 1,188 (20.2) |
|  | Household income (million yen), mean (SD) | 240.4 (156.9) | 253.4 (157.6) | 238.1 (155.1) |
|  | Employment, n (%) |  |  |  |
|  | Never | 769 (10.1) | 384 (9.1) | 592 (10.1) |
|  | Retired | 4,501 (59.1) | 2,493 (58.9) | 3.469 (59.0) |
|  | Current | 1,707 (22.4) | 1,083 (25.6) | 1,344 (22.9) |
|  | Marital status, n (%) |  |  |  |
|  | Married | 5,570 (73.2) | 3,250 (76.8) | 4,350 (73.8) |
|  | Single/others | 1,907 (25.1) | 930 (22.0) | 1,426 (24.3) |
|  | Living alone, n (%) | 1,032 (13.6) | 502 (11.9) | 734 (12.5) |
|  | Activities of daily living (independent), n (%) | 7,268 (95.5) | 4,090 (96.6) | 5,645 (96.0) |
|  | Population density (per square kilometer), mean (SD) | 4198.7 (3530.2) | 4,294.7 (3,578.6) | 2,995.7 (2,998.6) |
| Prior physical/cognitive health | |  |  |  |
|  | No natural teeth remaining, n (%) | 592 (7.8) | 274 (6.5) | 463 (7.9) |
|  | Self-rated health (excellent and good), n (%) | 6,404 (84.1) | 3,710 (87.7) | 4,988 (84.9) |
|  | Body mass index (kg/m2), mean (SD) | 23.0 (3.2) | 22.9 (3.1) | 23.0 (3.3) |
|  | Higher-level functional capacity, mean (SD) | 11.7 (1.7) | 11.9 (1.5) | 11.8 (1.6) |
|  | Self-reported hypertension, n (%) | 3,186 (41.9) | 1,728 (40.8) | 2,505 (42.6) |
|  | Self-reported diabetes, n (%) | 941 (12.4) | 504 (11.9) | 738 (12.6) |
|  | Self-reported dyslipidemia, n (%) | 1,014 (13.3) | 617 (14.6) | 794 (13.5) |
|  | Self-reported heart disease, n (%) | 730 (9.6) | 375 (8.9) | 553 (9.5) |
|  | Self-reported stroke, n (%) | 193 (2.5) | 93 (2.2) | 162 (2.8) |
|  | Self-reported respiratory disease, n (%) | 349 (4.6) | 175 (4.1) | 267 (4.5) |
| Prior mental health | |  |  |  |
|  | Depressive symptoms, mean (SD) | 2.9 (3.0) | 2.6 (2.9) | 2.9 (3.0) |
|  | Hopeless, n (%) | 1,129 (14.8) | 530 (12.5) | 851 (14.5) |
| Prior psychological well-being | |  |  |  |
|  | Happiness, mean (SD) | 7.4 (1.8) | 7.5 (1.7) | 7.4 (1.8) |
|  | Life satisfaction, n (%) | 6,267 (82.3) | 3,586 (84.7) | 4,867 (82.8) |
| Prior social well-being | |  |  |  |
|  | Participation in sports group, mean (SD) | 2.0 (1.6) | 2.1 (1.7) | 2.0 (1.6) |
|  | Participation in hobby group, mean (SD) | 2.2 (1.5) | 2.3 (1.6) | 2.2 (1.5) |
|  | Participation in senior citizens club, mean (SD) | 1.4 (0.9) | 1.3 (0.8) | 1.4 (0.9) |
|  | Participation in learning or cultural groups, mean (SD) | 1.3 (0.9) | 1.4 (0.9) | 1.3 (0.9) |
|  | Frequency of meeting friends, mean (SD) | 3.8 (1.6) | 3.8 (1.5) | 3.8 (1.5) |
|  | Number of friends seen within a month, mean (SD) | 3.6 (1.3) | 3.7 (1.3) | 3.6 (1.3) |
|  | Frequency of going out, mean (SD) | 5.7 (0.7) | 5.7 (0.6) | 5.7 (0.7) |
|  | Emotional social support, n (%) | 7,067 (92.8) | 3,963 (93.6) | 5,480 (93.2) |
|  | Instrumental social support, n (%) | 7,074 (92.9) | 3,994 (94.4) | 5,496 (93.5) |
| Pro-social/altruistic behaviors | |  |  |  |
|  | Volunteering, mean (SD) | 1.5 (1.1) | 1.5 (1.1) | 1.5 (1.0) |
|  | Sharing skills and experiences, mean (SD) | 1.3 (0.8) | 1.3 (0.9) | 1.3 (0.8) |
| Prior health behaviors | |  |  |  |
|  | Current smoking status, n (%) | 732 (9.6) | 387 (9.1) | 565 (9.6) |
|  | Frequency of meat and fish intake, mean (SD) | 5.3 (1.1) | 5.3 (1.1) | 5.3 (1.1) |
|  | Frequency of vegetables and fruits intake, mean (SD) | 6.1 (1.0) | 6.2 (0.9) | 6.2 (1.0) |
|  | Walking, mean (SD) | 2.4 (1.0) | 2.4 (1.0) | 2.4 (1.1) |
|  | Health screening, n (%) | 4,640 (61.0) | 2,753 (65.1) | 3,705 (63.0) |

Abbreviations: SD, standard deviation.


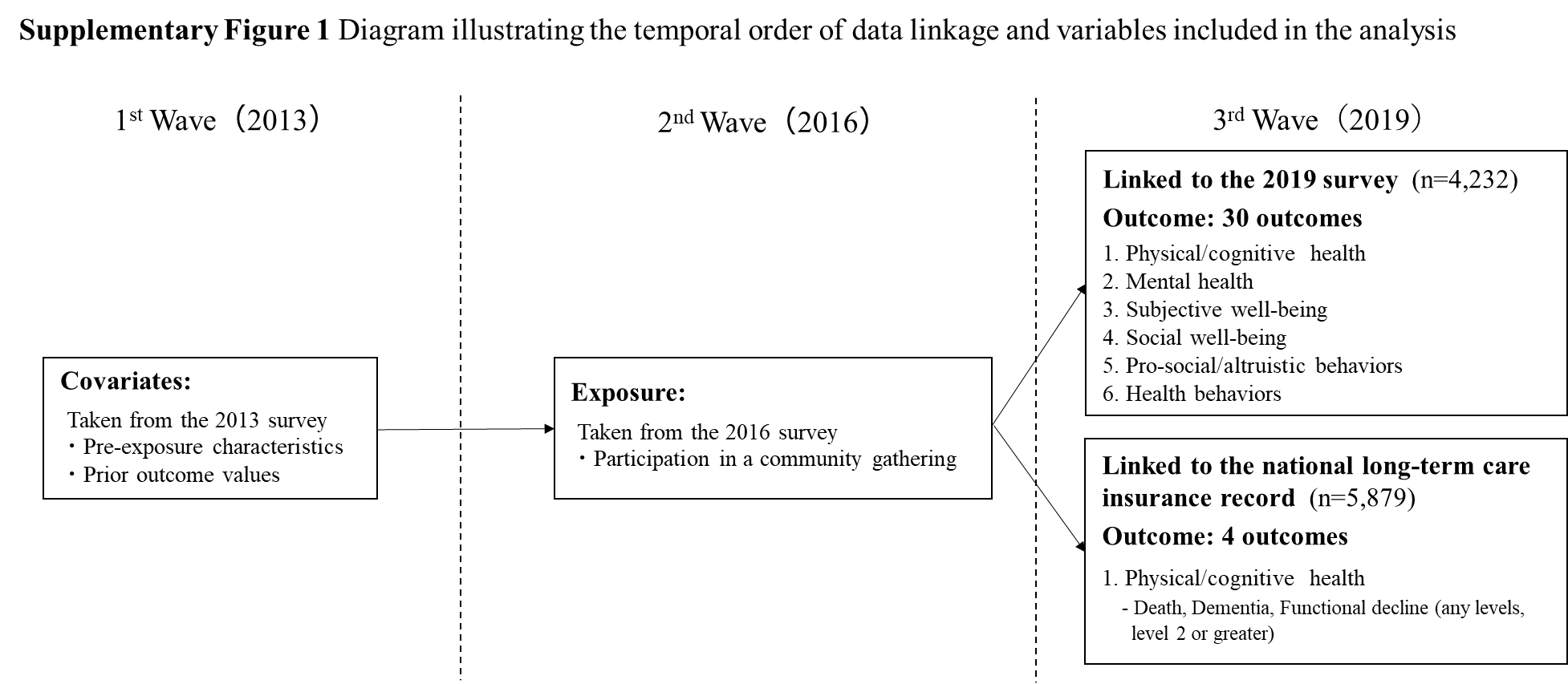

Supplement: igad084_suppl_Supplementary_Material [file igad084_suppl_supplementary_material.docx]
